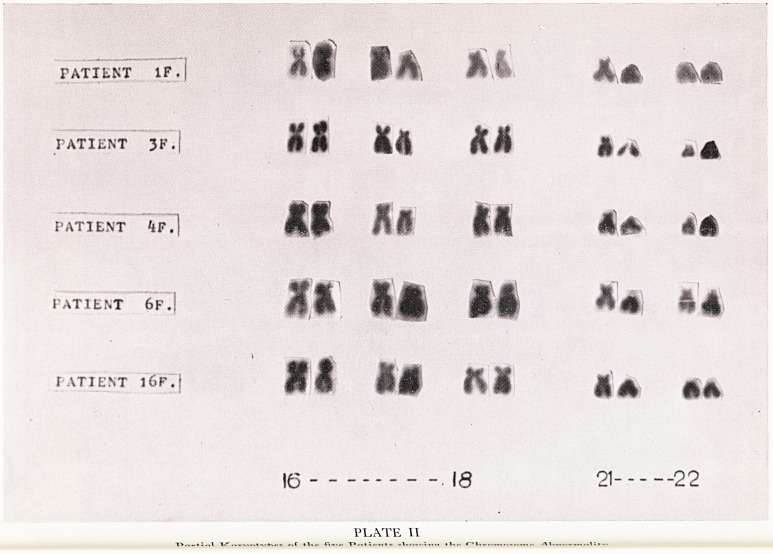# Chromosome Abnormality as a Possible Cause of Habitual Abortion

**Published:** 1965-01

**Authors:** Lydia Wingate


### CHROMOSOME ABNORMALITY AS A POSSIBLE CAUSE OF HABITUAL ABORTION

BY

LYDIA WINGATE, B.SC.

Spontaneous abortion is one of the commonest conditions to be met with in general and gynaecological practice. Treatment may be prolonged and is frequently disappointing. The loss of potential citizens is probably of the order of one in every three pregnancies. The woman is often deeply disappointed at the outcome of her pregnancy but when her loss is repeated on three or more occasions she may indeed feel desperate. Many doctors are of the opinion that treatment is of no great value apart from bed rest at the acute phase of the threat to abort. Most of these women together with their partners have had a prolonged series of investigations performed, at the end of which time no cause for their repeated abortions has been found.

It has been known for many years as a result of animal studies and breeding experiments, that there may be a genetic cause for recurrent foetal loss and that this may be transmitted or arise in a new generation as a mutation. A number of workers including Hertwig (1940), Koller and Auerbach (1941), Snell (1935), Tyler and Chapman (1948), and Waletzky and Owen (1942), have shown that minor alterations in chromosome morphology (induced in this case by irradiation) can cause foetal loss in rats and mice. The loss in these small animals does not take the form of habitual abortion—the dead foetuses are resorbed—but appears as a reduction in litter-size. What is important from the point of view of the human problem, however, is that this loss of litter-size is transmitted amongst the descendants of the irradiated animals. One of the more common of the chromosome abnormalities in these affected animals is a translocation in the germ cells. This originates from the breakage of a chromosome during meiosis, and the transfer of the broken fragment to another chromosome.

At the time that translocation occurs, some chromosome material may actually be lost, or there may be gene changes such as a localized loss or the production of a mutant gene; and any of these events may lead to the physiological impairment or death of the gamete or zygote (Hadorn, 1961). But if this does not occur, and the total genetic material of the germ cell is unimpaired, the translocation may be transmitted to subsequent generations.

Once the carrier-state for a translocation has become established, and the carrier mates with a normal individual, there is a possibility that non-viable offspring may result. In fact, four types of offspring may be produced as shown in Plate I. This is because the two pairs of chromosomes involved in the translocation may be distributed amongst the gametes in four different ways. Fertilization by a normal gamete then can produce four types of zygote. One of these is normal, one is genetically balanced but carries the translocation and two are genetically unbalanced and unlikely to survive (Schmid, 1962; Bateman, 1964).

It is reasonable to assume that translocation effects in man follow a pattern similar to that outlined above. Thus when habitual abortion occurs one might expect to find translocations in a proportion of the parents. In order to investigate this possibility blood chromosome studies have been carried out in Bristol since August 1963 on all couples when the wife's obstetric history presented the spontaneous loss of three or more successive early pregnancies.

An analysis of the peripheral blood leucocytes (cultured by a modification of the method described by Moorhead (1960)) was made in 22 couples and revealed chromosome aberrations in five of the women. In all cases the abnormality was associated with the chomosomes of groups 21/22 and 16/18 (Denver classification) as shown in Plate II. In each case one of the small acrocentric chromosomes shows abnormally long short arms. In Plate II, the chromosomes carrying the translocated materi[illegible] are in each case shown as the first members of the 21/22 group, and the chromosom[illegible] from which material has probably been lost are shown as the fourth members of th[illegible] 16/18 group. This chromosome aberration was found in at least 20 per cent of th[illegible] cells analysed. In all 44 subjects the modal number was 46 and the sex chromo-somes were normal. (Cells having a count of 45 were analysed and showed a rando[illegible] loss in all cases.)

TABLE 1

Chromosome Counts of the 5 Patients Showing a Chromosome Aberration

| Patient No. | Chromosome Counts | | | | | | Number of Cells | |
|---|---|---|---|---|---|---|---|---|
| | −45 | 45 | 46 | 47 | 47+ | Modal No. | Analysed | with Aberration |
| 1F | 2 | 4 | 51 | — | — | 46 | 55 | 25 |
| 3F | 6 | 12 | 106 | — | — | 46 | 118 | 50 |
| 4F | 1 | — | 43 | — | — | 46 | 43 | 21 |
| 6F | 2 | 8 | 100 | — | — | 46 | 108 | 22 |
| 16F | — | — | 40 | — | — | 46 | 40 | 31 |

Whether these translocations have arisen *de novo* or have been inherited can onl[illegible] be determined by family studies which it is hoped to carry out in all cases wher[illegible] abnormalities are found.

As the causes of abortion are probably multifactorial it is extremely unlikely tha[illegible] more than a small proportion will be due to a genetically unbalanced zygote. Howeve[illegible] it should be noted that using present day techniques, only the more obvious aberratio[illegible] will be detected, and therefore an apparently negative result does not rule out th[illegible] presence of an abnormality.

This work is being carried out with the aid of a research grant from the Unite[illegible] Bristol Hospitals, and the earlier findings were reported to a meeting of the Sout[illegible] Western Paediatric Society in December 1963.

I wish to express my thanks to Mr. R. G. Cousins of the Haematology Departme[illegible] of Bristol Royal Infirmary for his help with the blood cultures.

### REFERENCES

Bateman, A. J. (1964). *Lancet*, **i**, 310.

Hadorn, E. (1961). *Developmental Genetics and Lethal Factors*, Methuen & Co., London, pp. [illegible] and 254.

Hertwig, P. (1940). *Z.I.A.V.*, **79**, 1–27.

Koller, P. C. and Auerbach, C. A. (1941). *Nature*, **148**, 501–502.

Moorhead, P. S., Nowell, P. C., Mellman, W. S., Battips, D. M., and Hungerford, A. (1960[illegible] *Exp. Cell. Res.*, **20**, 613.

Schmid, W. (1962). *Cytogenet.*, **1**, 199.

Snell, G. D. (1935). *Genetics*, **20**, 545.

Tyler, W. J., and Chapman, A. B. (1948), *Genetics*, **33**, 565.

Waletzky, E. and Owen, R. D. (1942). *Genetics*, **27**, 173.

PLATE I

*Diagrammatic Representation to show the effect of a cross between a translocation heterozygote and a normal partner.*

PLATE II

## Figures and Tables

**PLATE I f1:**
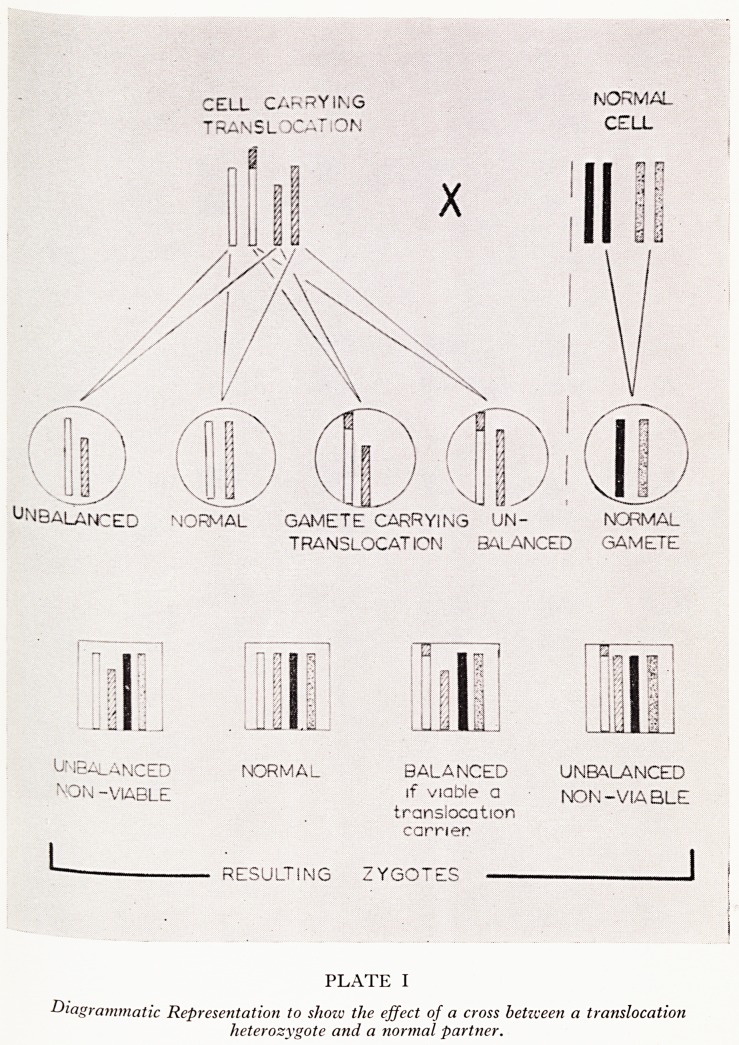


**PLATE II f2:**